# *In-Situ* Waviness Characterization of Metal Plates by a Lateral Shearing Interferometric Profilometer

**DOI:** 10.3390/s130404906

**Published:** 2013-04-12

**Authors:** María Frade, José María Enguita, Ignacio Álvarez

**Affiliations:** Department of Electrical Engineering, University of Oviedo, Campus de Viesques s/n, Gijón 33204, Spain; E-Mails: mfrade@isa.uniovi.es (M.F.); ialvarez@isa.uniovi.es (I.A.)

**Keywords:** waviness, profiling, lateral shearing interferometry

## Abstract

Characterizing waviness in sheet metal is a key process for quality control in many industries, such as automotive and home appliance manufacturing. However, there is still no known technique able to work in an automated in-floor inspection system. The literature describes many techniques developed in the last three decades, but most of them are either slow, only able to work in laboratory conditions, need very short (unsafe) working distances, or are only able to estimate certain waviness parameters. In this article we propose the use of a lateral shearing interferometric profilometer, which is able to obtain a 19 mm profile in a single acquisition, with sub-micron precision, in an uncontrolled environment, and from a working distance greater than 90 mm. This system allows direct measurement of all needed waviness parameters even with objects in movement. We describe a series of experiments over several samples of steel plates to validate the sensor and the processing method, and the results are in close agreement with those obtained with a contact stylus device. The sensor is an ideal candidate for on-line or in-machine fast automatic waviness assessment, reducing delays and costs in many metalworking processes.

## 1. Introduction

Producing sheet metal is one of the fundamental form processes in metalworking. Sheet metal has many different applications especially in the automotive and aerospace sectors, but also in the construction of everyday objects such as home appliances.

Sheet metal is usually produced in rolling mills and can be made of different materials, such as steel, aluminum, titanium or tin. In every case the finishing properties of the metal is a key point. In some cases it is important from the point of view of the final appearance of the product, in other cases for its tribological interactions (friction, erosion, abrasion, *etc.*); sometimes small cracks or defects may lead to client rejections or problems at later stages of the production process, *etc*.

Modern manufacturing requirements have boosted research on new methods for machining performance and quality inspection of final products, including dimensional control, detection of defects, and evaluation of surface waviness and roughness. Although there exist studies relating the dynamics in the rolling process and the final product surface characteristics [1], the surface finishing and waviness of sheet metal plates are still unpredictable in many machining processes [2,3], and the tools used in its characterization are becoming more and more sophisticated [4]. In this article we will focus on the measurement of the surface waviness.

Compared with roughness, waviness relates to the more widely spaced variations of the surface texture and is a key parameter in the quality of the final product. Waviness determines its resistance and lifespan, as well as the distribution of lubricant along its surface [5,6] or heat transfer properties [7]. Evaluating the waviness of a surface is also more challenging than evaluating roughness, as it requires the same depth precision in the measurements, but along much longer profiles (typically between 4 and 12.5 mm).

In addition to the usual contact profilometers, there exist specifically designed apparatus to measure waviness by contact methods [8]. To avoid the inconveniences of the needed contact between the sensor and the inspected surface, many optical techniques have been applied to both roughness and waviness evaluation, from white-light microscopy [9–12] to light scattering [13–16]. Other possibilities include laser triangulation [17–19], atomic force microscopes [20], or the use of synthetic holograms [21].

However, the high level of automation in the manufacturing processes demands fast and cost-effective systems able to operate *in-situ*. Interferometric techniques usually need controlled environments. Any point-wise technique would be slow in nature and would need controlled displacement systems in two directions to perform a surface scan. Microscopy and laser triangulation systems demand very short working distances to obtain the needed precision, which is an issue if applied *in-situ* or in automatic systems; some proposals include specific equipment for positioning control to minimize this problem. In addition, short working distances yield to small fields of view, so additional scanning along the profile direction together with matching algorithms are needed to obtain a profile with enough length to analyze waviness. Finally, light scattering techniques only estimate certain parameters of the waviness profile, such as average height variations. This does not suffice to characterize the properties of the surface in many cases [22].

In this article we investigate the applicability of a new optical sensor based on lateral-shearing interferometry for waviness analysis of metal sheets. This device, described in [23], is able to obtain distance profiles of 19 mm in a single shot, from a working distance greater than 90 mm, and a large depth of field (around 4 mm), with depth precisions of *σ* = 88 nm. Its characteristics make it ideal for its application in automatic surface inspection systems.

## 2. Materials and Methods

The interferometer is built around a Savart plate, which consists of two beam splitting plates made of birefringent material with their optic axes at 45° to the surface normal, rotated 90° with respect to each other. Light entering the Savart plate is divided in two components (ordinary and extraordinary) that exit parallel but with a small displacement (or shear) between them. An analyzer is used to bring both beams to the same state of polarization so they can interfere in the detection plane.

From any light reflecting point from a surface at a given distance, a spherical wave emerges, and the interference pattern carries information of the radius of this wavefront, or the distance to the surface.

### 2.1. Sensor Description

**Figure 1.**
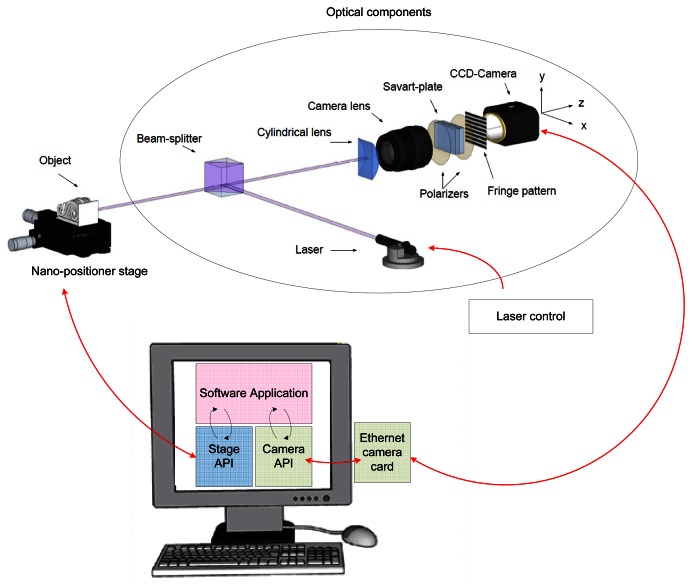
General setup of the prototype system.

Figure 1 shows the setup of the prototype. The system uses an *ImperX* IPX-2M30H-G charge-coupled device (CCD) camera with an active image area of 14.21 × 8 mm, a density of pixels of 1, 920 × 1, 080 (pixel size is 7.4 *μ*m), and 75 mm focal length lens. This provides an angle of view around 10°. With a typical working distance of 100 mm, we can obtain around 19 mm long profiles with a lateral resolution around 10 *μ*m per pixel.

The sample is mounted over a *Thorlabs NanoMax™* stage, controlled by the computer to perform surface scans.

The profile to be inspected is illuminated by projecting a laser line with a wavelength of 685 nm over the sample. The line thickness is 280–290 *μ*m when focused at 100 mm, and it is projected in the direction of the longer side of the CCD to maximize lateral resolution. A *SpeckleFree™* module manufactured by *Dyoptyka* is used to minimize speckle noise while keeping a high maximum frequency of acquisition (up to 500 kHZ).

In the detection branch a 50.8 mm focal length cylindrical lens is used to expand light in the direction perpendicular to the laser line. A calcite Savart plate with a total thickness of 7 mm sandwiched between two cross polarizers is placed between the camera lens and the CCD. This plate generates a shear of 0.53 mm for normal incidence.

#### 2.1.1. Fringe Formation

The formal derivation of fringe formation has been developed in depth in [23]. Here we will only outline the main results needed to follow the working principle of the sensor and the processing method.

In the direction of the profile (*x*-axis in Figure 1) the cylindrical lens has no effect, and the camera lens focuses the light onto the CCD. Therefore each column of the CCD contains the information of a small section of the profile (lateral resolution). We will consider this as one point of the profile.

**Figure 2.**
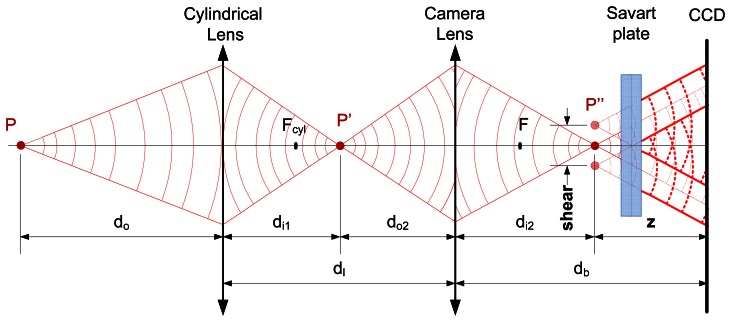
Schematic setup as observed from the direction perpendicular to the profile, *zy* plane (first appeared in [23], reprinted with permission).

The image formation in the direction perpendicular to the profile (*y*-axis) is defined by a two-lens system, as shown in Figure 2. The cylindrical lens generates an image of the light reflecting point *P* at *P*′ and the camera lens produces a second image of this point at *P*″, which is located at a distance *z* from the CCD. The wavefront is then duplicated with a lateral displacement (or shear) by the Savart plate, as if originated by two sources shown as the two red points at *P*″. Both wavefronts interfere in the CCD creating a pattern from which it is possible to calculate the distance *z*, and ultimately the distance *d_o_*.

The distance from the object to the sensor, *d_o_*, and from the image of the lens system to the CCD, *z*, are related by the opto-geometrical parameters of the system (the focal length of the camera lens, *F*, and the cylindrical lens *F_cyl_*, and the distances between components) as:

(1) $$z=d_{b}-\frac{F\;(-d_{o}\;d_{l}+F_{\text{cyl}\;}d_{l}+F_{\text{cyl}\;}d_{o})}{-d_{o}\;d_{l}+F_{\text{cyl}\;}d_{l}+F_{\text{cyl}\;}d_{o}+F\;d_{o}-F_{\text{cyl}\;}F}.$$

As a side note, it is important to observe that the distance *d_o_* increases when moving out of the center of the optical axis. Therefore for a flat profile at a distance *d* from the sensor, the distance *d_o_* follows an arc of circumference:

(2) $$d_{o}=\sqrt{d^{2}+h^{2}},$$

where *h* is the height above the optical axis of the point under consideration in the visualized profile.

As stated before, the signal recorded at each column of the CCD corresponds to one point in the line projected over the specimen under study. Therefore it is enough to formulate the expression of the interference signal as a function of the *y* coordinate in the detection plane. The frequency of the interference signal is proportional to the shear *s* introduced by the Savart plate, the distance *z*, and the wavelength λ of the illuminating source:

(3) $$I(y)=2\;I_{0}\left[1+\gamma_{0}\cos(2\pi \frac{s}{\lambda z}y)\right],$$

where *I*_0_ is the background intensity term and γ_0_ is the fringe visibility.

The shear can be calculated as a sum of terms, where the involved variables are: the ordinary and extraordinary refractive indexes of the crystal *n_o_* and *n_e_*, the total thickness of the Savart plate *t*, and the angle of incidence over its main section *i*. These expressions have been referred by Françon and Mallick [24]. The total shear can be approximated, by using the first two terms, as:

(4) $$\begin{array}{c} s=\sqrt{2}t/2\;\left[A+B\sin(i)\right],\text{where}:\\ A=\frac{n_{o}^{2}-n_{e}^{2}}{n_{o}^{2}+n_{e}^{2}},\\ B=\frac{n_{o}\;n_{e}}{{\left(\frac{n_{o}^{2}+n_{e}^{2}}{2}\right)}^{\frac{3}{2}}}-\frac{1}{n_{o}}. \end{array}$$

Both the angle of incidence and the distance *z* (or ultimately *d_o_*) determine the value of the fringe frequency. For this setup the angle of incidence carries information on the slopes over the profile with a much greater sensitivity than distance variations. Therefore, for flat objects like metal sheets, it is possible to separate both effects and reconstruct the profile of the object with high precision.

#### 2.1.2. Reconstruction Method

A Fast Fourier Transform (FFT) is applied to each column of the fringe pattern (typically 4096-point FFTs are used). The maximum peak of the frequency spectra is the frequency value of the of the interference signal (the cosine term in Equation (3)). From the fringe pattern we obtain a vector f with the frequency of fringes for each point *k* in the profile.

It is assumed that the surface under inspection is mainly flat, albeit for small irregularities which are outside the depth resolution of the system. In this case all the observed frequency deviations from the ideal shape are caused by changes in the slope of the surface and not by changes in the distance. From f and using Equation (3), we obtain a first approach of the profile z, which after filtering and applying a parabolic fit provides an estimation of the distance profile for a pure flat object at the same distanceẑ). Also Equations (1) and (2) can be used to obtain the distance to the sample *d_o_*.

Then, from Equations (3) and (4), it is possible to calculate the value of the angles of incidence i that generated changes in the frequency signal f due to small irregularities as:

(5) $$i_{k}=\arcsin\left(\frac{f_{k}\lambda {\hat{z}}_{k}-A}{B}\right).$$

Once the angle of incidence is known, the optical distance profile d to the camera lens (containing the small irregularities of the surface) is calculated for each element *k*, as:

(6) $$d_{k}=d_{k-1}-\frac{\Delta x_{p}\;[x-x_{p}+d_{b}\tan(i_{k})]}{d_{o}+d_{l}},$$

where Δ*x_p_* is the lateral resolution over the profile, and *x* and *x_p_* are the distances to the point *k* to the center of the profile in the CCD and in the original profile respectively.

**Figure 3.**
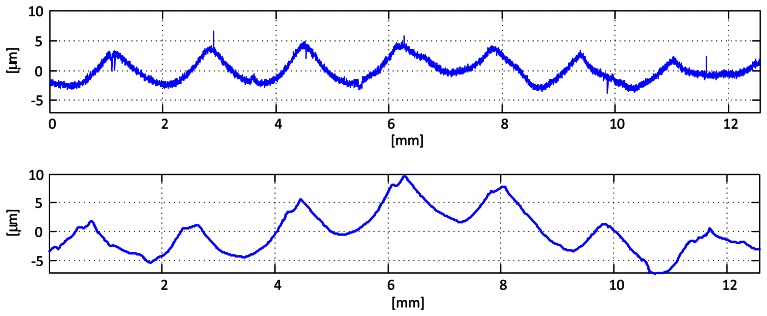
Examples of raw profiles obtained by the stylus contact instrument (**top**) and the optical profilometer (**down**).

The result of this process is shown in Figure 3, where surface profile of a sample obtained with a contact stylus instrument is compared with the one obtained with the optical sensor. The overall shape of the profile, showing the marks produced by the machining process, is correctly recovered. The small differences between both obtained profiles are mainly due to the impossibility of scanning exactly the same area and in the same direction, and the filtering of high frequencies caused by the thickness of the laser line (which results in averaging over a small area) and the lateral resolution of the sensor, which, for this working distance, is 10 *μ*m per pixel. Nonetheless, this filtering does not affect the evaluation of waviness, as one of the steps involved in the process is, in fact, removing the high spatial frequencies. Waviness measurement will be studied in the next section.

### 2.2. Waviness Measurement

Surface texture analysis has been standardized by the International Organization for Standardization (ISO) [25–28]. In industry, though, it is common to follow also the standards by the American Society for Mechanical Engineers (ASME) [29]. They both have slight differences regarding the calculation of the main statistical parameters. Other national standards are sometimes used, such as JIS B0601 and B0031 in Japan, NF E05-15 to 17 in France, DIN 47 68, 71, 75, 76 and 77 in Germany, and BS1134 in the United Kingdom.

#### 2.2.1. Characteristic Curves

To characterize the surface topology of a sample, the first step is obtaining the roughness and waviness profiles from the raw profile data produced by the sensor. To accomplish this, it is common to first remove the large-scale variations, or the *form*, of the profile (also sometimes called *flatness* or *curvature*) by removing a least squares fit on the data. The order of the fitting varies depending on the sample, but linear, square or cubic fits are most often used. Both ISO and ASME standards also recommend applying a low-pass filter to the raw data in order to remove measurement noise.

**Figure 4.**
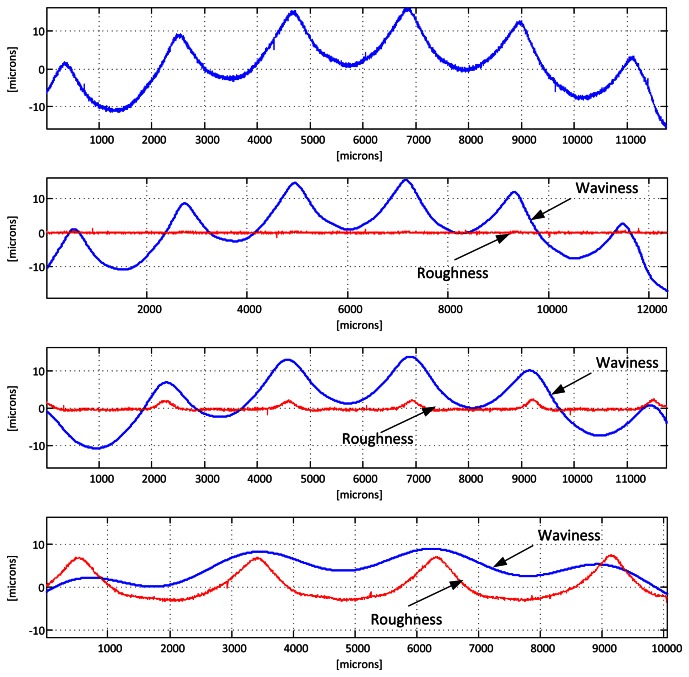
Separation of the waviness and roughness profiles from a sample. From top to bottom: a profile of a Rubert Type 130 reference specimen of a surface machined by horizontal milling (in this case the N8) obtained with a contact stylus instrument, and the waviness and roughness profiles obtained with values of λ*_c_* = 0.25, 0.8 and 2.5 respectively.

The second step consists in separating the longer wavelength variations of the height profile (*waviness*) from the shorter ones (*roughness*). ISO 11562 and 16610 standards impose the use of a Gaussian filter for this task, while ASME B46.1 standard contemplates a 2RC filter as an alternative option, as do most of the national standards, but DIN and JIS. In either case the cutoff wavelength of this filter (usually referred to as λ*_c_*) must be chosen, and its value is of utmost importance. It depends on several factors, including the finishing of the surface to be inspected (mainly its roughness) and the characteristics to evaluate (for instance the marks produced by the machining process), but its most common values are 0.25, 0.8 and 2.5 mm. The sampling length should be selected so that a good statistical analysis of the surface can be made. In most cases, five sample lengths are used for the analysis of roughness. Following this reasoning, at least 2–3 times this length should be used for the analysis of waviness. For a cutoff of 0.8 mm, this means a 8–12 mm profile. Figure 4 illustrates this process. As the Gaussian filter has a border effect, the first and last λ_c_/2 mm of the sample must be removed. This is why the valid profile length becomes shorter as the cutoff wavelength increases.

#### 2.2.2. Standardized Parameters

All the standards define a set of statistical parameters that can be calculated both over the roughness and the waviness profiles. In the national standards these parameters are calculated along all the evaluation length, while in the ISO standards the profile is first divided into sections (usually 5) that are analyzed separately. The final result is obtained by averaging.

Some of the most common parameters used to describe the surface topology are: *R_a_* (the average height variations over the base line), *R_q_* (quadratic deviation of height variations),*R_p_* and *R_v_* (highest peak and lowest valley), *R_t_* (peak to valley height), *R_z_* (average height), *R_ku_* and *R_sk_* (kurtosis and skewness of the height distribution), *t_p_* (the profile bearing ratio), or *H_tp_* (the height between two points on the bearing ratio curve at specified values of 20% and 80%).

When the parameters are calculated for the waviness profile, they are usually denoted as *W_a_, W_q_, W_p_, etc.*[25,28,29].

## 3. Results and Discussion

**Figure 5.**
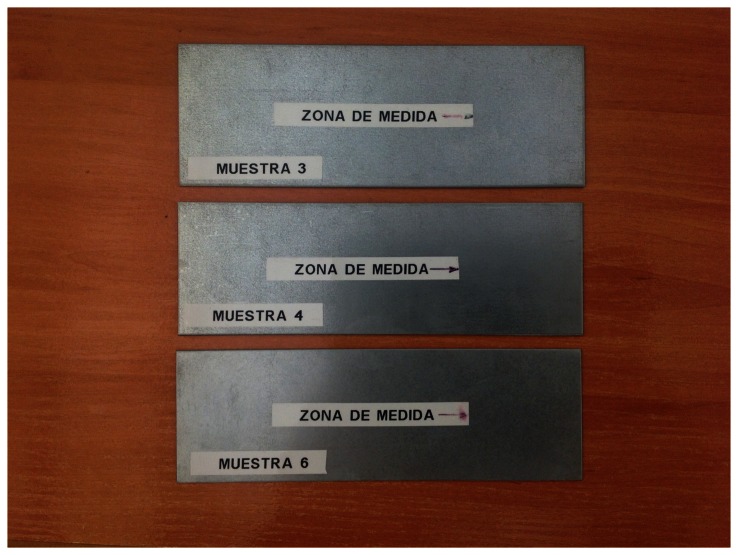
Examples of the steel plates used for testing.

**Figure 6.**
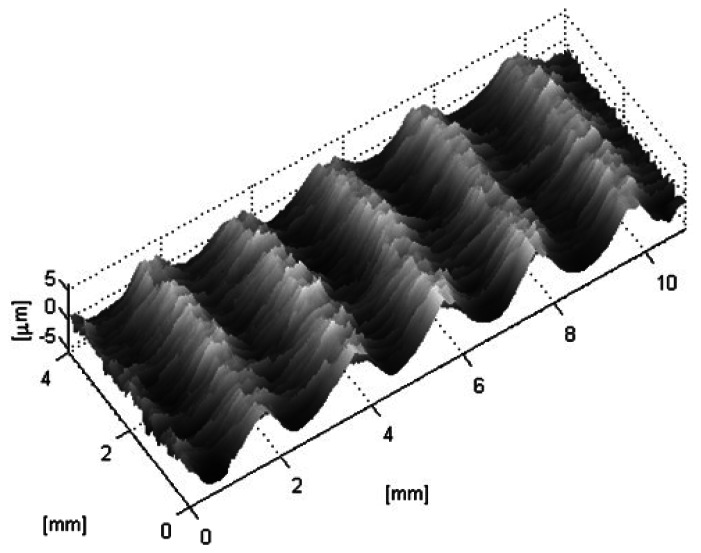
3D surface reconstruction of a Rubert Type 130 reference specimen of a surface machined by horizontal milling (in this case the N7), where the marks of the machining process are clearly seen.

The test set consists of eight samples of steel plates produced by rolling under different conditions, like those shown in Figure 5, and two samples of the Rubert set 130 finished by horizontal milling (N7, *R_a_* =1.6 *μ*m and N8, *R_a_* =3.2 *μ*m). All the specimens were analyzed both with the optical sensor and with a contact stylus for comparison. With the optical device, more than 70 profiles of 19 mm were scanned for each sample, in a strip 4 mm wide. Figure 6 shows a surface plot of one of these samples.

Three different profiles for each sample were scanned with the contact stylus instrument in the same zone and direction. Instead of using the built-in processing capabilities of the stylus, the raw data was exported and processed with the same algorithms than the data from the optical sensor, to avoid any possible difference in the implementation of the methods. All the calculations follow the recommendations in the ISO standards.

**Figure 7.**
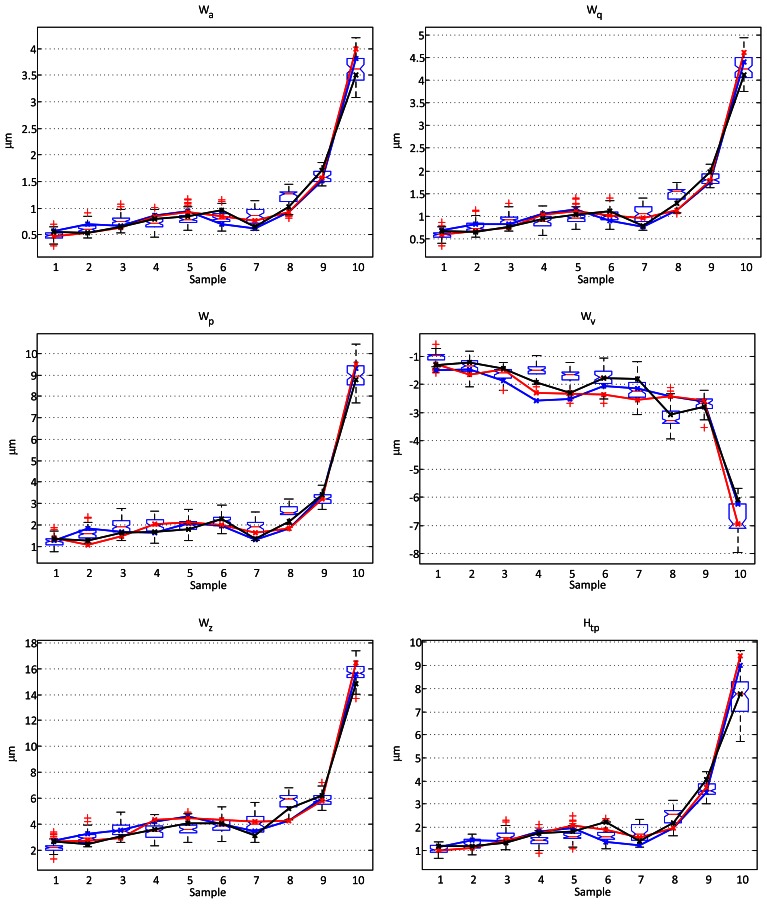
Comparison of results between a contact stylus profilometer and the proposed optical sensor for each of of the waviness parameters *W_a_, W_q_, W_p_, W_v_, W_z_*, and *H_tp_* using a cutoff wavelength of 0.25 mm. The boxplots represent the dispersion of the parameters calculated using the optical sensor data from more than 70 profiles of each sample. The continuous lines represent the parameters calculated over three different profiles obtained from the contact stylus instrument.

**Figure 8.**
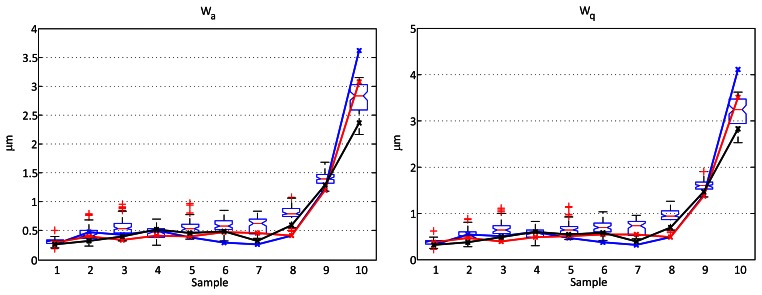
Comparison of results between a contact stylus profilometer and the proposed optical sensor for the waviness parameters *W_a_* and *W_q_* using a cutoff wavelength of 0.8 mm. The boxplots represent the dispersion of the parameters calculated using the optical sensor data from more than 70 profiles of each sample. The continuous lines represent the parameters calculated over three different profiles obtained from the contact stylus instrument. The rest of the parameters have been omitted in this case, as the results were similar to those in Figure 7.

In every case the raw profile was first filtered with a cubic fit to remove the form. Then a Gaussian filter was used to extract the waviness profiles. Two different cutoff wavelengths were used: 0.25 and 0.8 mm. Finally, the main parameters were calculated for all the profiles. Though it is quite common to suppose normal distribution of the value of the parameters for different profiles of the same sample, the non-parametric boxplot method is used for comparison, without making any assumptions of the underlying statistical distribution. The values calculated over the profiles obtained with the stylus are used as references and plotted using a solid line superimposed to the boxplot. Figures 7 and 8 show the comparative results.

### 3.1. Analysis of the Results

For the cutoff wavelength of 0.25 mm all the values of the measured *W_a_* and *W_q_* parameters for the contact stylus instrument fall inside the range of the measurements made with the optical instrument, and only one value (3.3%) and two values (6.6%) fall outside limits (though still very close) for the *H_tp_* and *W_z_* respectively. The number of values from the stylus falling outside the limits for the *W_v_* and *W_p_* parameters is higher (10% and 20% respectively). Their values, however, are not far from those obtained with the optical instrument, and these differences are not totally unexpected due to the high sensitivity of this parameters to occasional peaks or valleys in a given profile, and even measurement noise. This cutoff wavelength of 0.25 mm is the lowest that can be used in this configuration of the sensor, as it is near the thickness of the projected laser line. The measurement for any point in the profile is, in fact, an average of the distances of all points illuminated by the laser. A thinner laser line would permit using smaller cutoff wavelengths.

When the selected cutoff wavelength is 0.8 mm, the number of values outside the limits of the boxes gets higher (a 20% in the *R_a_* parameter). However, they are still quite close to these limits and are comparable to what are considered as outliers in the boxplot, except for sample 10. As this occurs when increasing the wavelength of the cutoff filterr, it is most probably caused by small differences in the direction of scanning, as will be discussed later.

Kurtosis (*W_ku_*)and skewness (*W_sk_*) were also analyzed but, as they are higher order surface moments, they show heavy variabilities, and the results were very similar for all the steel sheet specimens in the experiment; hence they are not very meaningful for this study. Kurtosis showed variations in the range from 1.5 to 3.5 for all the samples and only 4 reference values fall outside limits, though they are still very close (the maximum distance from a boxplot limit being 0.3). Skewness was also similar for all samples, with only 2.5% of the measurements above 0.6 and 1.4% below −0.6 and all the samples, except 9 and 10, showing results that extend from positive to negative values. This is also expected for random surfaces (a skewness factor in the range [−0.5, 0.5] is considered to represent a centered distribution).

Samples 9 and 10 (Rubert 130 N7 and N8 horizontal milling specimens) were the only exceptions, showing much lower variations in the kurtosis and skewness factors. It is interesting to note that skewness for these specimens was always positive and in the range [0.1, 0.6] in most of the observations (99.18%). All the results obtained with the stylus instrument are inside the limits.

The effect of increasing the cutoff wavelength in this case is just that the dispersion decreases but does not change the comparative results.

### 3.2. Variability of the Parameters over Different Profiles in the Same Specimen

It is not possible to measure exactly the same profile every time over a given sample. As the working conditions in the process change constantly due to many reasons (dynamic phenomena, vibrations, temperature changes, *etc.*), it is quite common to obtain different values for different measurements. Rodriguez *et al.*[30] make an analysis of this variability considering a Gaussian distribution to resolve the deviance. These authors report variations of ±28.4% with a confidence level of 95% (±2σ) in the measurement of *R_a_* over different profiles in a sample with a mean of 0.653 *μ*m. This illustrates the importance of scanning several profiles (an area) and giving not only the mean value of the parameters but also their variations (for instance as the value of the standard deviation).

In the experiments we observed a similar amount of variability in the parameters when calculated over the different profiles we acquire for each specimen, though a bit higher most probably due to the different nature of the forming process. Table 1 shows the variability of the *W_a_* parameter calculated with a cutoff wavelength of 0.25 mm, both as the standard deviation of the measurements and as a percentage over the mean value with a confidence level of 95%. Similar results are obtained for a cutoff wavelength of 0.8 mm.

For the samples produced by rolling, this variability is in the range from ±26% to ±35%, while for the samples produced by horizontal milling (from the Rubert set) it decreases to about ±16%.

The same authors also analyze the effect of the direction of scanning. Most of the machining processes have a directional effect and the values of the roughness and waviness parameters vary dramatically when the measurements are made parallel or perpendicular to this direction. In some types of machine processes, such as grinding, this difference can be as high as a factor of 5.

When analyzing waviness this effect is very relevant, as even slight differences in the direction of scanning (which are very difficult to avoid) change the wavelength of the main marks produced by the machining process and thus how they are affected by the Gaussian filter for a given cutoff wavelength. Such an alignment difference is the most probable cause of the outlier for sample 10 in Figure 8, where a cutoff of 0.8 mm was used.

## 4. Conclusions

Results indicate that the optical profilometer based on lateral shearing interferometry described in Section 2.1 accurately serves the purpose of characterizing the waviness of sheet metal plates. The experimental results are in close agreement with those obtained with a contact stylus instrument. The optical device has many characteristics that make it an excellent candidate for in-process or in-machine integration: its common path nature makes it quite insensitive to vibrations and other environmental conditions, it is able to operate from a large working distance (above 90mm for this prototype), and it has a large depth of field (about 4 mm). The precision of the sensor with this configuration is *σ* < 88 nm with an accuracy of 40 nm and a repeatability of *σ* = 10 nm, as reported in [23].

This unique combination of large working distance, high depth resolution and aperture, high depth of field, and acquisition speed, makes the device especially adequate for automatic waviness assessment, even with objects in movement.

The smallest cutoff wavelength usable with the current system was 0.25 mm, imposed by the thickness of the projected laser line. This suffices for most applications. Nevertheless, if a 0.08 cutoff wavelength is needed, the best option would be reducing the working distance. This should allow the generation of a much thinner line with only a change in the line generator and the camera lenses.

The system is able to characterize surfaces with *W_a_* up to approximately 4 *μ*m. Surfaces with higher *W_a_* may not be correctly reconstructed as they usually have high steeps, which may pose a problem to the system (light might not enter the sensor). Occasional peaks or valleys of surfaces with even low *W_a_* may also be filtered for the same reason, but they would be removed by the Gaussian filter anyway, so they do not affect the calculation of the waviness parameters.

The instrument performed the acquisition of the profiles while the sample was moved laterally. The data corresponding to a whole profile is obtained in single camera acquisition at frame rate, so the whole set of profiles for a given area is obtained in a few seconds. The data corresponding to the different samples was obtained without the need of recalibrating or reconfiguring the system whatsoever (simply changing samples on the stage), and without the need to pause the movement to perform the acquisition. This is very important for waviness characterization, where the need of analyzing an area and not a single profile, and providing not just the mean result but also its variability along different profiles, has been shown.

The average processing time for each profile is about 0.3 seconds in an Intel® Core™ i7 processor with 4 physical cores, mainly dominated by the calculation of the 1920 FFTs. There is still room, however, for further optimizations, for instance by rewriting the core calculations in assembly language.

## Figures and Tables

**Table 1.** Variability of the *W_a_* parameter when calculated over different profiles for each sample with a cutoff wavelength of 0.25 mm.

Sample	mean *W_a_* (*μm*)	standard deviation	Variability at 95%
**1**	0.485	0.084	± 34.6%
**2**	0.619	0.106	± 34.2%
**3**	0.762	0.111	± 29.1%
**4**	0.722	0.112	± 31.0%
**5**	0.809	0.128	± 31.6%
**6**	0.824	0.122	± 29.6 %
**7**	0.845	0.149	± 35.3 %
**8**	1.209	0.157	± 26.0 %
**9**	1.593	0.123	± 15.4%
**10**	3.638	0.297	± 16.3%
